# Near‐complete genome assembly and annotation of the yellow drum (*Nibea albiflora*) provide insights into population and evolutionary characteristics of this species

**DOI:** 10.1002/ece3.4778

**Published:** 2018-12-11

**Authors:** Zhaofang Han, Wanbo Li, Wen Zhu, Sha Sun, Kun Ye, Yangjie Xie, Zhiyong Wang

**Affiliations:** ^1^ Key Laboratory of Healthy Mariculture for the East China Sea, Ministry of Agriculture, Fisheries College Jimei University Xiamen China; ^2^ Laboratory for Marine Fisheries Science and Food Production Processes Qingdao National Laboratory for Marine Science and Technology Qingdao China

**Keywords:** annotation, genome assembly, nucleotide diversity, yellow drum (*Nibea albiflora*)

## Abstract

Yellow drum (*Nibea albiflora*) is an important fish species in capture fishery and aquaculture in East Asia. We herein report the first and near‐complete genome assembly of an ultra‐homologous gynogenic female yellow drum using Illumina short sequencing reads. In summary, a total of 154.2 Gb of raw reads were generated via whole‐genome sequencing and were assembled to 565.3 Mb genome with a contig N_50_ size of 50.3 kb and scaffold N_50_ size of 2.2 Mb (BUSCO completeness of 97.7%), accounting for 97.3%–98.6% of the estimated genome size of this fish. We further identified 22,448 genes using combined methods of ab initio prediction, RNAseq annotation, and protein homology searching, of which 21,614 (96.3%) were functionally annotated in NCBI nr, trEMBL, SwissProt, and KOG databases. We also investigated the nucleotide diversity (around 1/390) of aquacultured individuals and found the genetic diversity of the aquacultured population decreased due to inbreeding. Evolutionary analyses illustrated significantly expanded and extracted gene families, such as myosin and sodium: neurotransmitter symporter (SNF), could help explain swimming motility of yellow drum. The presented genome will be an important resource for future studies on population genetics, conservation, understanding of evolutionary history and genetic breeding of the yellow drum and other *Nibea* species.

## 1 INTRODUCTION

**Figure 1:**
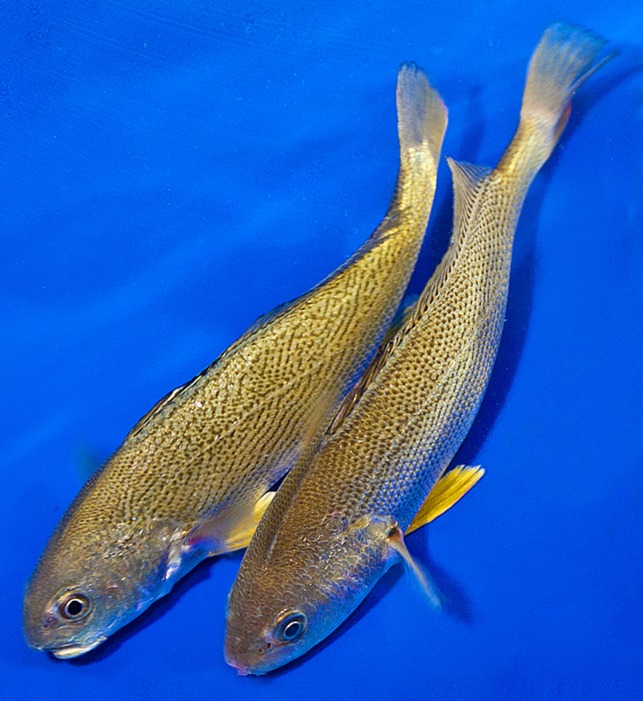
The yellow drum (*Nibea albiflora*). The picture of the yellow drum was provided by Shuqiu Xie (Mindong Fishery Research Institute of Fujian Province)

Yellow drum (*Nibea albiflora*; Figure 1) belongs to the Sciaenidae, one of the largest family of the Perciforms, and is naturally distributed in southern Japan and East China Sea (Takita, 1974; Xu et al., 2017). The yellow drum can grow to ~44 cm in length and ~1.5 kg in weight (Shunpei & Kazuo, 1980), reaching sex maturity at age of 2 ~ 3 years. It inhabits preferentially in coastal waters with depth of 70 ~ 80 meters and has seasonal migratory patterns. Yellow drum, together with large yellow croaker (*Larimichthys crocea*, another species in the Sciaenidae family ranking first in annual production among all marine fish aquaculture in China (Guo and Zhao, 2017)), account for an important part of sea food consumption in China, especially in east China. Yellow drum shares similar external characteristics, meat quality and flavors with large yellow croaker. Now, yellow drum is expected to partially replace the market of the large yellow croaker, which suffers from the shortage of germplasm resource due to overfishing and severe bacterial and virus diseases in aquaculture (Chen, Lin, & Wang, 2003; Han et al., 2016). Recently, the wild population of the yellow drum is declining due to overfishing and aquaculture of this fish is expanding to meet market needs (Cheng, Xu, Jin, & Wang, 2011). It is worthwhile to conduct researches on wild population conservation and genetic improvement of this fish. The genus *Nibea* consists of eleven recognized species, widely distributed in the Indo‐West Pacific oceans (Lo et al., 2015; Lo, Liu, Nor, & Chen, 2017). Up to date, no reference genome for the *Nibea* species is present, hampering the studies on conservation and genetic investigation of these species.

In the present study, we took efforts to assemble the first and near‐complete reference genome of an ultra‐homologous gynogenic female yellow drum using Illumina short sequencing reads. Based on the assembled genome, the genome‐wide nucleotide diversity of cultured yellow drum has been investigated, which can reflect the degree of inbreeding of aquaculture populations. Besides, the phylogenetic relationships of yellow drum with other teleosts were previously described (Lo et al., 2015, 2017); however, there is not a study conducted so far for phylogenetic analysis based on whole‐genome data, and we thus inferred the phylogeny with the yellow drum and other teleosts. And analysis of expanded and contracted gene families can aid to investigate the evolution of specific characteristics of this fish. In conclusion, the near‐complete reference genome of the yellow drum we provided laid a solid foundation for the conservation, evolutionary studies, and genetic improvement of this species.

## 2 MATERIALS AND METHODS

### 2.1 Sample, library construction, and sequencing

An ultra‐homozygous female yellow drum homozygous at 11 highly polymorphic microsatellite loci (Supporting Information Table S1) was produced through two generations of gynogenesis in a mariculture farm in Ningde, Fujian province, China. Genomic DNA was extracted from its muscle tissue using the traditional phenol–chloroform isolation method (Sambrook & Russell, 2006). DNA concentration was measured using Qubit 2.0 Fluorometer (Life Technologies, CA, USA). During the whole‐genome shotgun sequencing, four paired‐end libraries (two with insert sizes of 300 bp and two with insert sizes 450 bp) were constructed using the Illumina TruSeq Nano method (Illumina, CA, USA). Three mate‐pair libraries with long‐insert sizes of 2 kb, 5 kb, and 10 kb were generated using the Nextera Mate Pair Sample Preparation Kit (Illumina, CA, USA). In brief, 4 μg of genomic DNA was fragmented and tagged with biotinylated mate‐pair junction adapters. Subsequently, the strand displacement reaction was performed and the DNA was purified with AMPure XP beads. The 2 kb, 5 kb, and 10 kb DNA fragments were selected using a 0.75% cassette for the BluePippin (Sage Science, MA, USA). DNA was recovered and ligated overnight at 30°C following the manufacturer's protocol. After incubation and heat inactivation, exonuclease was added to remove non‐circularized DNA. The samples were subsequently end repaired, A‐tailed, and adapter ligated, and enrichment was achieved with 10 rounds of PCR. All prepared libraries were sequenced (150 bp × 2) on a HiSeq X platform (Illumina, USA) at Novogene Bioinformatics Technology Co., Ltd (Beijing, China). For quality control, FASTQC (Andrews, 2013) was used to check quality of the raw reads; Trimmomatic (Bolger, Lohse, & Usadel, 2014) was employed to trim the adapter sequences and to remove low‐quality bases (Phred score <20) of the paired‐end reads, and reads shorter than 50 bp were discarded. All reads from mate‐pair libraries were trimmed to a length of 50 bp to avoid potentially spanning the junctions of the DNA circularization.

### 2.2 Genome assembly and evaluation

Platanus v1.2.4 (Kajitani et al., 2014) was employed to assemble the genome sequence of *N. albiflora* through three steps: contig assembling, scaffold construction, and gap closing. Although Platanus was originally developed for assembling highly heterozygous genomes, it is also with better performance in constructing super large scaffolds compared to other assemblers, irrespective of heterozygosity of the genomes (Kajitani et al., 2014). First, all the paired‐end sequencing data with short inserts were used to construct *de Bruijn* graphs with automatically optimized k‐mer sizes. Then, the mate‐pair sequencing reads were used to link contigs into scaffolds. At gap‐closing step, all sequencing reads were utilized to fill intra‐scaffold gaps using paired‐end information, where one end of a pair uniquely mapped to a scaffold and the other end located within a gap.

The quality of the assembled genome was evaluated as follows: (a) The sequencing reads with short inserts (300 bp and 450 bp) were realigned to the assembled genome using BWA v0.7.17 (Li & Durbin, 2009). (b) RNAseq data of a pool of multiple tissues were aligned to the genome assembly using STAR v2.5.3a (Dobin et al., 2013). (c) BUSCO (Benchmarking Universal Single‐Copy Orthologs) v3.0 (Simao, Waterhouse, Ioannidis, Kriventseva, & Zdobnov, 2015) was employed to assess the completeness of the assembly using database of actinopterygii_odb9.

### 2.3 Repeat‐content identification and classification

To identify known repeats and transposable elements (TEs), RepeatMasker v4.0.6 (Smit, Hubley, & Green, 2013–2015) was used to align the genome assembly against the Repbase TE library with the default parameters (Jurka et al., 2005). In addition, a de novo repeat library was constructed using RepeatModeler v1.0.4 (Smit & Hubley, 2008–2015) with the genomic sequences of *N. albiflora*. The RepeatMasker was employed again to identify repeat elements using the de novo repeat library. The repeat elements identified from Repbase library and de novo library were merged together and masked for further analysis.

### 2.4 Gene prediction and annotation

The repeat‐masked genome was utilized to predict gene structures through ab initio gene prediction, protein homology‐based prediction, and transcript evidence. Augustus v3.2.3 (Stanke, Diekhans, Baertsch, & Haussler, 2008), GeneMark‐ET v4.32 (Lomsadze, Burns, & Borodovsky, 2014), and Braker v1.9 (Hoff, Lange, Lomsadze, Borodovsky, & Stanke, 2016) were applied for the ab initio gene prediction. For Augustus v3.2.3 (Stanke et al., 2008), the known gene structures of zebrafish and the RNAseq assembled transcripts (Han, Xiao, Li, Ye, & Wang, 2018) were used in model parameter training, and the optimum parameters were obtained after two rounds of training. The homology‐based gene prediction was done by aligning protein sequences of *Homo sapiens *(human), *Danio rerio *(zebrafish), *Takifugu rubripe *(pufferfish), *Oryzias latipes *(medaka), *Gasterosteus aculeatus *(three‐spined stickleback), *Larimichthys crocea *(large yellow croaker), and *Dicentrarchus labrax *(European seabass) to the *N. albiflora* genome assembly using tblastn (E‐value: 1e−5) (Altschul et al., 1997). Aligned sequences were submitted to Exonerate v2.2.0 (Slater & Birney, 2005) for defining precise splicing sites and exons. In addition, transcripts assembled from RNAseq, which was sequenced on an Illumina HiSeq 2,500 platform (2 × 125 bp) with pooled library of 11 tissues (Han et al., 2018), were employed to build comprehensive transcripts database and identify open reading frame (ORF) using PASA v2.1.0 program (Haas et al., 2003). After removing the genes with coding region length shorter than 150 bp, a consensus gene set was created from the genes of the three different sources using EVM (Evidence Modeler) v1.1.1 (Haas et al., 2008). All predicted genes were then aligned to NCBI nr, trEMBL, SwissProt, and KOG databases for function annotation using blastx (E‐value: 1e−5) (Camacho et al., 2009).

### 2.5 Calculation of nucleotide diversity

The nucleotide diversity of yellow drum was measured using four randomly sampled fish (two males and two females) from the aquaculture populations in Fujian Province in 2016. DNA was extracted from dorsal fins of the four fish using TIANamp Genomic DNA Kit (TIANGEN, Beijing, China). And paired‐end sequences (150 bp × 2) were generated on an Illumina HiSeq X platform. The resequencing data of the four individuals were sequenced at 32.8 ~ 38.4 × coverage and were aligned to the genome assembly of the yellow drum using BWA v0.7.17 (Li & Durbin, 2009). Duplicated reads were subsequently removed, and aligned reads around insertions/deletions (Indels) were realigned using GATK v3.8.0 (Mckenna et al., 2010). Platypus v0.8.1 (Rimmer et al., 2014) was utilized to call SNPs and small Indels (≤10 bp) on the refined bam files. Nucleotide diversity was calculated by counting the frequency of heterozygous sites in high‐quality variants (genotype quality > 60 and 10 < depth < 100) in each individual.

### 2.6 Phylogeny and gene family comparison

The protein sequences of 11 species, including *Homo sapiens* (human), *Gallus gallus* (chicken), *Lepisosteus oculatus* (spotted gar), *Tetraodon nigroviridis* (spotted green pufferfish), *Xiphophorus maculatus* (platyfish), *Oreochromis niloticus* (tilapia), *D. rerio *(zebrafish), *T. rubripe *(pufferfish), *O. latipes *(medaka), *G. aculeatus *(three‐spined stickleback), and *L. crocea *(large yellow croaker), were obtained from Ensembl. The longest sequences extracted from each gene were aligned with each other among the 12 species using ClustalW v2.0 (Larkin et al., 2007). Based on the alignment information, the OrthoMCL v2.0.9 (Li, Stoeckert, & Roos, 2003) was employed to obtain pairs of one‐to‐one protein‐coding orthologs, and the orthologs were used to construct the phylogenetic tree using maximum likelihood estimation (MLE) method with MEGA7 (Kumar, Stecher, & Tamura, 2016). The divergence time for *N. albiflora* and other vertebrates was estimated based on the time‐calibrated divergence between human and chicken (320 million years ago (MYA), obtained from the TimeTree database (Hedges, Dudley, & Kumar, 2006)).

In gene family analysis, protein sequences coded by the longest isoform of each gene from all the 12 species were aligned to each other exhaustively using blastp (E‐value: 1e−5). Based on the alignments, gene clustering and gene copy number of a certain gene family within each species were obtained using OrthoMCL v2.0.9 (Li et al., 2003). And node time was estimated via r8s software (Sanderson, 2003). Finally, gene family expansion and contraction analyses were conducted by CAFÉ (Bie, Cristianini, Demuth, & Hahn, 2006).

## 3 RESULTS AND DISCUSSION

### 3.1 Genome size estimation and assembling

We generated a total of 154.2 Gb of raw data (~260 × coverage) for the gynogenetic yellow drum on an Illumina HiSeq X platform. After quality trimming and filtering, we retained 119.8 Gb data for genome assembling (Table 1). A subset of the cleaned sequencing reads of 300 bp insert library (~29.2 Gb) was used to obtain a frequency distribution of 17‐, 21‐, 25‐, and 29‐mers of the yellow drum genome through Jellyfish v2.4.0 (Marcais & Kingsford, 2011) (Supporting Information Figure S1). The resulting histograms were explored to estimate the size, repeat content, and heterozygosity of the yellow drum genome via the GenomeScope software (Vurture et al., 2017). The estimated genome size of the yellow drum was between 573.2 Mb (17‐mers) and 581.0 Mb (29‐mers) (Supporting Information Table S2). The results were consistent with the estimated genome size (595.7 Mb) using flow cytometry (Cao, Zheng, Wang, Liu, & Cai, 2015). The 29‐mer analysis indicates that the yellow drum genome possesses a low level of repeat content (45.6 Mb, 7.9%).

**Table 1:** Summary statistics of the whole‐genome sequencing data

Library	Library type	Insert size (bp)	Reads number	Total base (bp)	Reads number after trimming	Total bases after trimming (bp)
DES00946	Paired‐end	300	214,014,344	32,102,151,600	213,149,506	31,435,660,732
DES00947	Paired‐end	300	175,073,346	26,261,001,900	174,401,308	25,728,984,413
DES00948	Paired‐end	450	158,969,122	23,845,368,300	157,188,702	22,982,429,030
DES00949	Paired‐end	450	166,828,960	25,024,344,000	164,607,364	24,031,348,725
DEL00758	Mate‐pair	2,000	104,631,768	15,694,765,200	103,664,548	5,231,588,400
DEL00757	Mate‐pair	5,000	116,137,928	17,420,689,200	114,947,454	5,806,896,400
DEL00775	Mate‐pair	10,000	91,955,960	13,793,394,000	90,954,304	4,597,798,000

**Figure 2:**
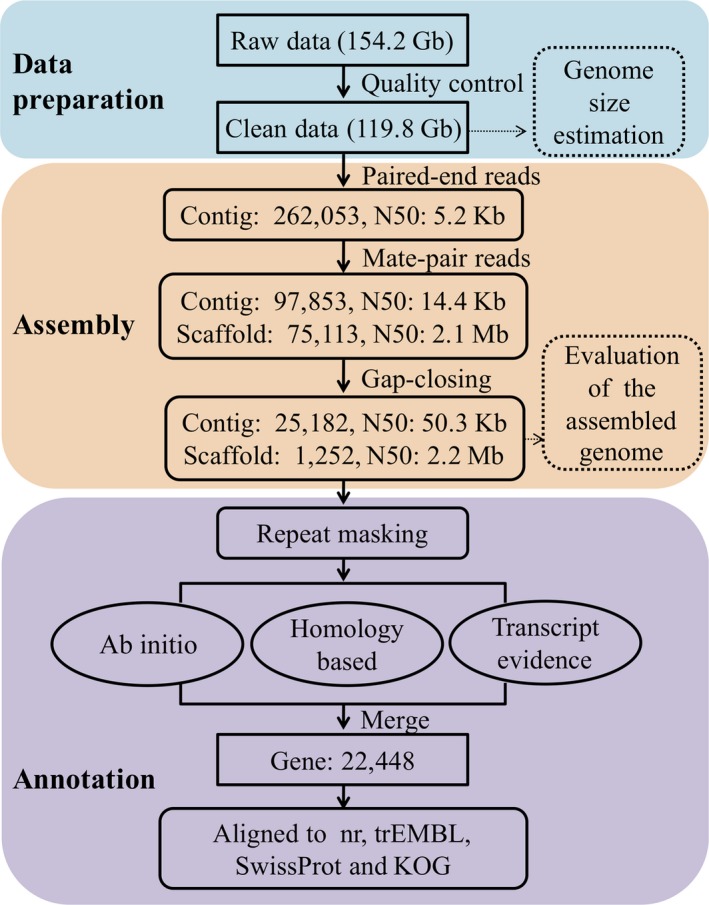
The flow chart depicting the whole‐genome sequencing, assembly, and annotation

As shown in Figure 2, all the paired‐end reads were first assembled into contigs with N_50_ of 5.2 kb. Then, scaffolding was conducted using the mate‐pair reads to link contigs into 75,113 scaffolds. And the contig N_50_ reached 14.4 kb, and the scaffold N_50_ was 2.1 Mb. Finally, the gap closing increased the contig N_50_ to 50.3 kb, and the scaffold (>2 kb) N_50_ was 2.2 Mb (Table 2). The final assembled genome of the *N. albiflora *is 565.3 Mb in length with 1,252 scaffolds, which accounts for 97.3%–98.6% of the k‐mer estimated genome size and hence indicates a near‐complete genome assembly.

**Table 2:** Summary statistics of the *Nibea albiflora* genome assembly

Assembly	Contig	Scaffold
Size (bp)	557,190,737	565,299,463
GC content (%)	42.4	41.8
Number	25,182	1,252
N_50_ size (bp)	50,300	2,254,189
Shortest (bp)	301	2,002
Longest (bp)	641,168	13,214,368
Average length (bp)	22,127	451,517
N bases (bp)	0	8,026,962

Different methods were used to evaluate the quality of our assembled genome. Libraries of paired‐end sequencing data of 300 bp and 450 bp were realigned to the assembly, total mapping rates were 99.4% and 99.3%, and mapping ratios of properly paired reads were 95.4% and 91.2%, respectively. We further aligned transcriptomic data to the assembly, demonstrating a mapping ratio of 92.2%. The gene completeness assessment shows that 4,478 (97.7%) among 4,584 Benchmarking Universal Single‐Copy Orthologs (BUSCOs) were completely detected by BUSCO annotation, including 4,362 complete and single‐copy, 116 complete and duplicated, 39 fragmented, and 67 missing orthologs (Supporting Information Table S3). The assessment analyses again suggested that the genome assembly of the yellow drum was sufficiently accurate and near complete.

### 3.2 Repeat sequences in the yellow drum

Structure‐based searching with Repbase identified 813,598 repeat elements in the yellow drum, which constituted approximately 78.1 Mb (13.8%) of the assembled genomes. And the dominant type is simple repeat elements, accounting for 2.8% of the yellow drum genome (Table 3). The proportion of repeated elements in the yellow drum was much lower than those found for other teleosts, such as medaka (Kasahara et al., 2007) (17.5% of 700 Mb), large yellow croaker (Ao et al., 2015) (18.1% of 728 Mb), and Atlantic cod (Star et al., 2011) (25.4% of 830 Mb), which might be one of the reasons that the yellow drum owns a smaller‐size genome than those species.

**Table 3:** Summary of repeat elements identified in the *Nibea albiflora* genome

Repeat element	Fragments	Total length (bp)	% of genome
SINE	20,650	2,761,049	0.5
LINE	40,857	7,549,448	1.3
LTR element	16,278	3,870,965	0.7
DNA element	84,443	12,838,672	2.2
RC element	4,504	1,205,596	0.2
Small RNA	1,578	127,371	0.02
Simple repeat	380,033	15,842,155	2.8
Low complexity	40,161	2,123,227	0.4
Unclassified	225,094	31,820,793	5.5
Total	813,598	78,139,276	13.7

### 3.3 Functional annotation of predicted gene

Based on integrated methods of ab initio prediction, protein‐based homology and transcript evidence, we obtained a final gene set containing 22,448 genes with an average gene length of 12,764 bp, coding sequence length of 1,844 bp, and an average of 13.4 exons per gene (Table 4). In more detail, 21,587, 21,092, 20,054, and 19,859 genes were annotated in nr, trEMBL, SwissProt, and KOG, respectively. A total of 21,614 (96.3%) genes obtained at least one hit in the four databases. The predicted genes and annotation rates were comparable to same family species such as large yellow croaker (Ao et al., 2015) (25,401 annotated genes), medaka (Kasahara et al., 2007) (20,141 non‐redundant genes), and Atlantic cod (Star et al., 2011) (22,154 protein‐coding genes).

**Table 4:** Summary statistics for gene prediction for *Nibea albiflora* genome

		Gene number	Average gene length (bp)	Average CDS length (bp)	Average exons per gene
De novo	Augustus	25,718	11,121	1,754	12.8
	GeneMark‐ET	59,067	3,277	767	5.4
	Braker	27,331	10,700	1,699	12.2
Homolog	*Homo sapiens*	51,671	16,589	936	6.2
	*Danio rerio*	33,864	17,462	1,241	7.6
	*Takifugu rubripe*	45,545	19,485	1,557	10.0
	*Oryzias latipes*	21,873	13,336	1,287	8.1
	*Gasterosteus aculeatus*	25,185	13,059	1,342	8.7
	*Larimichthys crocea*	23,825	15,024	1,619	9.3
	*Dicentrarchus labrax*	23,815	14,520	1,477	8.4
Transcriptome	PASA	6,746	9,826	1,589	15.4
Merge	Evidence Modeler	22,448	12,764	1,844	13.4

### 3.4 Nucleotide diversity of aquacultured yellow drum

Yellow drum aquaculture has existed for more than 20 years in east China. To get an idea of the population diversity of the aquacultured yellow drum, we measured the nucleotide diversity of four individuals randomly sampled from the aquaculture populations. As a result, the average nucleotide diversity of the four individuals was 0.26% (~1/390; range: 0.23%–0.27%), which was comparable to or even higher than that of wild populations of the previously reported marine fish (1/309 in herring (Barrio et al., 2016), 1/435 in coelacanth (Amemiya et al., 2013), 1/500 in cod (Star et al., 2011), and 1/700 in stickleback (Jones et al., 2012)). This indicates that the current aquaculture population of the yellow drum could be still genetically diverse. During 2004–2005, the nucleotide diversity of the wild‐caught yellow drum was estimated using 33 polymorphic SNPs from three locations along the coastal regions of the Yellow Sea and the East China Sea, and it ranged from 0.30% to 1.16% (Qingdao), from 0.33% to 1.27% (Zhoushan), and from 0.42% to 1.56% (Xiamen), respectively (Han, Gao, Yanagimoto, & Sakurai, 2008). The nucleotide diversity in wild population is much higher than that found in this study, suggesting the aquaculture population might have suffered from inbreeding.

### 3.5 Phylogenetic analysis and gene family

**Figure 3:**
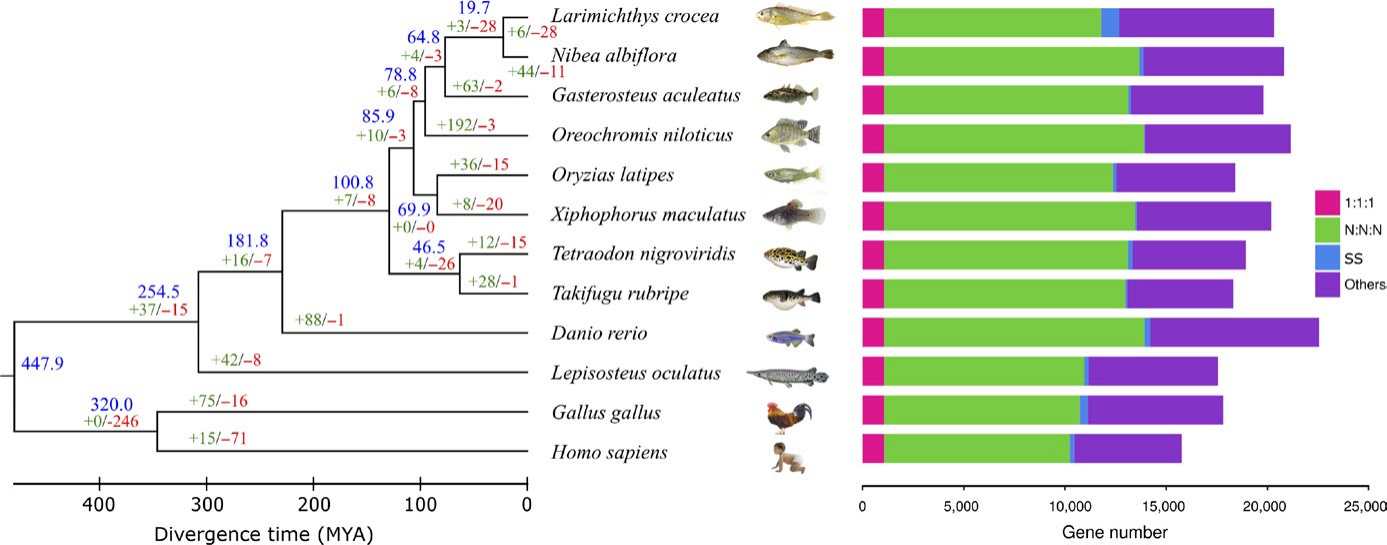
Phylogenetic tree and orthologous genes in *Nibea albiflora *and 11 other vertebrates. Blue numbers in the phylogenetic tree indicate the divergence time (MYA, million years ago), and the green and red numbers represent the expanded and contracted gene families, respectively. The histogram shows different types of orthologous relationships. “1:1:1” means universal single‐copy genes; “N:N:N” means orthologs exist in all genomes; “SS” means species‐specific genes; and “Others” means orthologs that do not fit into the other categories

To determine the evolutionary position of *N. albiflora*, we performed systematic genome comparisons among *N. albiflora* and 11 other vertebrates. The phylogenetic tree was finally constructed based on 1,070 pairs of one‐to‐one protein‐coding orthologs using the MLE method. The yellow drum is evolutionarily close to the large yellow croaker, and the divergence time between them is about 19.7 MYA (Figure 3). Among all other teleosts, the three‐spined stickleback is a close sister group with the yellow drum and large yellow croaker (MYA: 64.8), which is consistent with the previous phylogenetic analysis of Sciaenidae and Gasterosteiformes (Ao et al., 2015).

Furthermore, 44 significantly expanded and 11 contracted gene families (*p* < 0.01) were identified via comparing the gene families of the yellow drum with that of other vertebrates used in the phylogenetic analysis (Supporting Information Table S4). Expansion and contraction analyses can provide clues for inference of underlying genetic basis of specific physiological characteristics. For example, myosin family genes (such as *MYH6*, *MYH7,* and *MYH11*) were significantly expanded in the genome of yellow drum (33) compared with the large yellow croaker (12). Myosin acts as functional protein and structural protein, and is directly involved in many biological functions such as muscle contraction, cardiac regulation, cell movement, and signal transduction in animals (Fu & Zhang, 2008). Yellow drum is closer to large yellow croaker in phylogeny (Figure 3). However, it has been noted that yellow drum has better swimming ability than the large yellow croaker in the mariculture practice, which could be partially attributed to the expansion of family of myosin genes. Besides, sodium: neurotransmitter symporter (SNF) family genes (such as *SLC6A1*, *SLC6A6*, *SLC6A8*, *SLC6A11*, *SLC6A12,* and *SLC6A13*) were also expanded in yellow drum in contrast to large yellow croaker. Those genes are essential for the release, re‐uptaking, and recycling of neurotransmitters at synapses (Attwell & Bouvier, 1992). *SLC6A1*, *SLC6A11,* and *SLC6A13*, widely distributed in brain, encode sodium‐dependent transporters that uptake gamma‐aminobutyric acid (GABA) (Zhou et al., 2012). Defects in those genes may result in epilepsy, behavioral problems, or intellectual problems. *SLC6A6*, *SLC6A8,* and *SLC6A12*, expressed abundantly in the skeletal muscle, play key roles in optimal uptake and osmotic regulation (Borden, Smith, Gustafson, Branchek, & Weinshank, 2002; Sora et al., 1994). Those expanded genes might be also important for motility and osmotic regulation in the yellow drum. The detailed mechanism should be investigated in the future.

## 4 CONCLUSIONS

Here, we report the first genome assembly of the yellow drum, which has been demonstrated to be highly accurate and near complete. The results showed that our strategy of using a homozygous individual (gynogen) and better assembly algorithm (e.g., Platanus) in de novo genome assembling was powerful with just Illumina short reads. The near‐complete genome and its annotation allowed us to perform population genetics and evolutionary analyses in the yellow drum. These resources will also be necessary in conservation efforts and genetic breeding in this fish species. In addition, this study will illuminate undiscovered genetic characteristics of *Nibea* genus in the future studies on these species. Furthermore, the assembly is fragmented in contigs and is not ideally used for chromosome‐scale assembling. Hence, the genome still needs to be improved in the future with advanced technologies (e.g., long‐read sequencing and optical mapping).

## CONFLICT OF INTERESTS

The authors declare that they have no competing interests.

## AUTHOR CONTRIBUTIONS

Z.H. and W.L. performed all the analyses and wrote the manuscript. W.Z. and S.S. collected the samples and performed the wet lab experiments. K.Y. and Y.X. conducted the gynogenesis and raised the experimental fish population. Z.W. conceived and supervised the study, and revised the manuscript. All authors agreed on the final version of this manuscript.

## DATA ACCESSIBILITY

All whole‐genome shotgun sequencing data are publicly available in the NCBI SRA (accession no.: PRJNA432345). Raw sequence reads of resequencing of four individuals and transcriptome data are available in NCBI under accession no.: PRJNA432345, PRJNA431723 and PRJNA359138. The assembled genome has been deposited into European Nucleotide Archive (ENA) under accession no. PRJEB24302.

## References

[ece34778-bib-0001] Altschul, S. F. , Madden, T. L. , Schaffer, A. A. , Zhang, J. , Zhang, Z. , Miller, W. , & Lipman, D. J. (1997). Gapped BLAST and PSI‐BLAST: A new generation of protein database search programs. Nucleic Acids Research, 25, 3389–3402. 9254694 10.1093/nar/25.17.3389 PMC146917

[ece34778-bib-0002] Amemiya, C. T. , Alfoldi, J. , Lee, A. P. , Fan, S. , Philippe, H. , Maccallum, I. , … Lindblad‐Toh, K. (2013). The African coelacanth genome provides insights into tetrapod evolution. Nature, 496, 311–316. 23598338 10.1038/nature12027 PMC3633110

[ece34778-bib-0003] Andrews, S. (2013). FastQC a quality control tool for high throughput sequence data.

[ece34778-bib-0004] Ao, J. , Mu, Y. , Xiang, L.‐X. , Fan, D. , Feng, M. , Zhang, S. , … Chen, X. (2015). Genome sequencing of the perciform fish *Larimichthys crocea* provides insights into molecular and genetic mechanisms of stress adaptation. PLoS Genetics, 11, e1005118. 25835551 10.1371/journal.pgen.1005118 PMC4383535

[ece34778-bib-0005] Attwell, D. , & Bouvier, M. (1992). Cloners quick on the uptake. Current Biology, 10, 541–543. 10.1016/0960-9822(92)90024-5 15336049

[ece34778-bib-0006] Barrio, A. M. , Lamichhaney, S. , Fan, G. , Rafati, N. , Pettersson, M. , Zhang, H. , … Andersson, L. (2016). The genetic basis for ecological adaptation of the Atlantic herring revealed by genome sequencing. eLife, 5, e12081. 27138043 10.7554/eLife.12081 PMC4854517

[ece34778-bib-0007] Bolger, A. M. , Lohse, M. , & Usadel, B. (2014). Trimmomatic: A flexible trimmer for Illumina sequence data. Bioinformatics, 30, 2114–2120. 24695404 10.1093/bioinformatics/btu170 PMC4103590

[ece34778-bib-0008] Borden, L. A. , Smith, K. E. , Gustafson, E. L. , Branchek, T. , & Weinshank, R. L. (2002). Cloning and expression of a betaine/GABA transporter from human brain. Journal of Neurochemistry, 64, 977–984. 10.1046/j.1471-4159.1995.64030977.x 7861179

[ece34778-bib-0009] Camacho, C. , Coulouris, G. , Avagyan, V. , Ma, N. , Papadopoulos, J. , Bealer, K. , & Madden, T. L. (2009). BLAST+: Architecture and applications. BMC Bioinformatics, 10, 421. 20003500 10.1186/1471-2105-10-421 PMC2803857

[ece34778-bib-0010] Cao, K. , Zheng, J. , Wang, Z. , Liu, X. , & Cai, M. (2015). Genome size and physical length of chromosomes in *Nibea albiflora* . South China Fisheries Science, 11, 65–70.

[ece34778-bib-0011] Chen, X. , Lin, K. B. , & Wang, X. W. (2003). Outbreaks of an iridovirus disease in maricultured large yellow croaker, *Larimichthys crocea* (Richardson), in China. Journal of Fish Diseases, 26, 615–619. 14653319 10.1046/j.1365-2761.2003.00494.x

[ece34778-bib-0012] Cheng, Y.‐Z. , Xu, T.‐J. , Jin, X.‐X. , & Wang, R.‐X. (2011). Complete mitochondrial genome of the yellow drum *Nibea albiflora* (Perciformes, Sciaenidae). Mitochondrial DNA, 22, 80–82. 10.3109/19401736.2011.624602 21985406

[ece34778-bib-0013] De Bie, T. , Cristianini, N. , Demuth, J. P. , & Hahn, M. W. (2006). CAFE: A computational tool for the study of gene family evolution. Bioinformatics, 22, 1269–1271. 16543274 10.1093/bioinformatics/btl097

[ece34778-bib-0014] Dobin, A. , Davis, C. A. , Schlesinger, F. , Drenkow, J. , Zaleski, C. , Jha, S. , … Gingeras, T. R. (2013). STAR: Ultrafast universal RNA‐seq aligner. Bioinformatics, 29, 15–21. 23104886 10.1093/bioinformatics/bts635 PMC3530905

[ece34778-bib-0015] Fu, G. , & Zhang, J. (2008). Progress and prospect on the studies of fish myosin heavy chain and its genes. Letters in Biotechnology, 19, 306–309.

[ece34778-bib-0016] Guo, Y. , & Zhao, W. (2017). China fishery statistical yearbook. Beijing, China: China Agricultural Press.

[ece34778-bib-0017] Haas, B. J. , Delcher, A. L. , Mount, M. S. , Wortman, J. R. , Smith, R. K. , Hannick, L. I. , … White, O. (2003). Improving the Arabidopsis genome annotation using maximal transcript alignment assemblies. Nucleic Acids Research, 31, 5654–5666. 14500829 10.1093/nar/gkg770 PMC206470

[ece34778-bib-0018] Haas, B. J. , Salzberg, S. L. , Zhu, W. , Pertea, M. , Allen, J. E. , Orvis, J. , … Wortman, J. R. (2008). Automated eukaryotic gene structure annotation using EVidenceModeler and the Program to Assemble Spliced Alignments. Genome Biology, 9, 1–22. 10.1186/gb-2008-9-1-r7 PMC2395244 18190707

[ece34778-bib-0019] Han, F. , Zhang, Y. , Zhang, D. , Liu, L. , Tsai, H. J. , & Wang, Z. (2016). The Rab5A gene of marine fish, large yellow croaker (*Larimichthys crocea*), and its response to the infection of Cryptocaryon irritans. Fish & Shellfish Immunology, 54, 364–373. 27108380 10.1016/j.fsi.2016.04.025

[ece34778-bib-0020] Han, Z. Q. , Gao, T. X. , Yanagimoto, T. , & Sakurai, Y. (2008). Genetic population structure of *Nibea albiflora* in Yellow Sea and East China Sea. Fisheries Science, 74, 544–552.

[ece34778-bib-0021] Han, Z. , Xiao, S. , Li, W. , Ye, K. , & Wang, Z. Y. (2018). The identification of growth, immune related genes and marker discovery through transcriptome in the yellow drum (*Nibea albiflora*). Genes & Genomics, 40(8), 881–891. 10.1007/s13258-018-0697-x 30047113

[ece34778-bib-0022] Hedges, S. B. , Dudley, J. T. , & Kumar, S. (2006). TimeTree: A public knowledge‐base of divergence times among organisms. Bioinformatics, 22, 2971–2972. 17021158 10.1093/bioinformatics/btl505

[ece34778-bib-0023] Hoff, K. J. , Lange, S. , Lomsadze, A. , Borodovsky, M. , & Stanke, M. (2016). BRAKER1: Unsupervised RNA‐Seq‐based genome annotation with GeneMark‐ET and AUGUSTUS. Bioinformatics, 32, 767–769. 26559507 10.1093/bioinformatics/btv661 PMC6078167

[ece34778-bib-0024] Jones, F. C. , Grabherr, M. , Chan, Y. F. , Russell, P. , Mauceli, E. , Johnson, J. , … Kingsley, D. M. (2012). The genomic basis of adaptive evolution in threespine sticklebacks. Nature, 484, 55–61. 22481358 10.1038/nature10944 PMC3322419

[ece34778-bib-0025] Jurka, J. , Kapitonov, V. V. , Pavlicek, A. , Klonowski, P. , Kohany, O. , & Walichiewicz, J. (2005). Repbase update, a database of eukaryotic repetitive elements. Cytogenetic and Genome Research, 110, 462–467. 16093699 10.1159/000084979

[ece34778-bib-0026] Kajitani, R. , Toshimoto, K. , Noguchi, H. , Toyoda, A. , Ogura, Y. , Okuno, M. , … Itoh, T. (2014). Efficient de novo assembly of highly heterozygous genomes from whole‐genome shotgun short reads. GenomeResearch, 24, 1384–1395. 10.1101/gr.170720.113 PMC4120091 24755901

[ece34778-bib-0027] Kasahara, M. , Naruse, K. , Sasaki, S. , Nakatani, Y. , Qu, W. , Ahsan, B. , … Kohara, Y. (2007). The medaka draft genome and insights into vertebrate genome evolution. Nature, 447, 714. 17554307 10.1038/nature05846

[ece34778-bib-0028] Kumar, S. , Stecher, G. , & Tamura, K. (2016). MEGA7: Molecular Evolutionary Genetics Analysis version 7.0 for bigger datasets. Molecular Biology and Evolution, 33, 1870–1874. 27004904 10.1093/molbev/msw054 PMC8210823

[ece34778-bib-0029] Larkin, M. A. , Blackshields, G. , Brown, N. P. , Chenna, R. , McGettigan, P. A. , McWilliam, H. , … Higgins, D. G. (2007). Clustal W and Clustal X version 2.0. Bioinformatics, 23, 2947–2948. 17846036 10.1093/bioinformatics/btm404

[ece34778-bib-0030] Li, H. , & Durbin, R. (2009). Fast and accurate short read alignment with Burrows‐Wheeler transform. Bioinformatics, 25, 1754–1760. 19451168 10.1093/bioinformatics/btp324 PMC2705234

[ece34778-bib-0031] Li, L. , Stoeckert, C. J. , & Roos, D. S. (2003). OrthoMCL: Identification of Ortholog Groups for Eukaryotic Genomes. Genome Research, 13, 2178–2189. 12952885 10.1101/gr.1224503 PMC403725

[ece34778-bib-0032] Lo, P.‐C. , Liu, S.‐H. , Chao, N. L. , Nunoo, F. K. , Mok, H.‐K. , & Chen, W.‐J. (2015). A multi‐gene dataset reveals a tropical New World origin and Early Miocene diversification of croakers (Perciformes: Sciaenidae). Molecular Phylogenetics and Evolution, 88, 132–143. 25848970 10.1016/j.ympev.2015.03.025

[ece34778-bib-0033] Lo, P.‐C. , Liu, S.‐H. , Nor, S. A. M. , & Chen, W.‐J. (2017). Molecular exploration of hidden diversity in the Indo‐West Pacific sciaenid clade. PLoS ONE, 12, e0176623. 28453569 10.1371/journal.pone.0176623 PMC5409148

[ece34778-bib-0034] Lomsadze, A. , Burns, P. D. , & Borodovsky, M. (2014). Integration of mapped RNA‐Seq reads into automatic training of eukaryotic gene finding algorithm. Nucleic Acids Research, 42, e119. 24990371 10.1093/nar/gku557 PMC4150757

[ece34778-bib-0035] Marcais, G. , & Kingsford, C. (2011). A fast, lock‐free approach for efficient parallel counting of occurrences of k‐mers. Bioinformatics, 27, 764–770. 21217122 10.1093/bioinformatics/btr011 PMC3051319

[ece34778-bib-0036] Mckenna, A. , Hanna, M. , Banks, E. , Sivachenko, A. , Cibulskis, K. , Kernytsky, A. , … DePristo, M. A. (2010). The Genome Analysis Toolkit: A MapReduce framework for analyzing next‐generation DNA sequencing data. Genome Research, 20, 1297–1303. 20644199 10.1101/gr.107524.110 PMC2928508

[ece34778-bib-0037] Rimmer, A. J. , Phan, H. , Mathieson, I. , Iqbal, Z. , Twigg, S. R. F. , WGS500 Consortium , … Lunter, G. (2014). Integrating mapping‐, assembly‐ and haplotype‐based approaches for calling variants in clinical sequencing applications. Nature Genetics, 46, 912–918. 25017105 10.1038/ng.3036 PMC4753679

[ece34778-bib-0038] Sambrook, J. , & Russell, D. W. (2006). Purification of nucleic acids by extraction with Phenol:Chloroform. CSH Protocols, 2006, 4455. 10.1101/pdb.prot4455 22485786

[ece34778-bib-0039] Sanderson, M. J. (2003). r8s: Inferring absolute rates of molecular evolution and divergence times in the absence of a molecular clock. Bioinformatics, 19, 301–302. 10.1093/bioinformatics/19.2.301 12538260

[ece34778-bib-0040] Shunpei, K. , & Kazuo, N. (1980). On the age and growth of *Nibea albiflora* . Bulletin of the Japanese Society of Scientific Fisheries, 46, 139–143.

[ece34778-bib-0041] Simao, F. A. , Waterhouse, R. M. , Ioannidis, P. , Kriventseva, E. V. , & Zdobnov, E. M. (2015). BUSCO: Assessing genome assembly and annotation completeness with single‐copy orthologs. Bioinformatics, 31, 3210–3212. 10.1093/bioinformatics/btv351 26059717

[ece34778-bib-0042] Slater, G. , & Birney, E. (2005). Automated generation of heuristics for biological sequence comparison. BMC Bioinformatics, 6, 31. 15713233 10.1186/1471-2105-6-31 PMC553969

[ece34778-bib-0043] Smit, A. , Hubley, R. (2008–2015). *RepeatModeler Open‐1.0* . Retrieved from http://www.repeatmasker.org (13 March 2017, date last accessed).

[ece34778-bib-0044] Smit, A. , Hubley, R. , Green, P. (2013–2015). *RepeatMasker Open‐4.0* . Retrieved from http://www.repeatmasker.org (6 January 2017, date last accessed).

[ece34778-bib-0045] Sora, I. , Richman, J. , Santoro, G. , Wei, H. , Wang, Y. , Vanderah, T. , … Yamamura, H. I. (1994). The cloning and expression of a human creatine transporter. Biochemical and Biophysical Research Communications, 204, 419–427. 7945388 10.1006/bbrc.1994.2475

[ece34778-bib-0046] Stanke, M. , Diekhans, M. , Baertsch, R. , & Haussler, D. (2008). Using native and syntenically mapped cDNA alignments to improve de novo gene finding. Bioinformatics, 24, 637–644. 18218656 10.1093/bioinformatics/btn013

[ece34778-bib-0047] Star, B. , Nederbragt, A. J. , Jentoft, S. , Grimholt, U. , Malmstrøm, M. , Gregers, T. F. , … Jakobsen, K. S. (2011). The genome sequence of Atlantic cod reveals a unique immune system. Nature, 477, 207–210. 21832995 10.1038/nature10342 PMC3537168

[ece34778-bib-0048] Takita, T. (1974). Studies on the early life history of *Nibea albiflora* (Richardson) in Ariake Sound. Bulletin of the Faculty of Fisheries Nagasaki University, 38, 1–55.

[ece34778-bib-0049] Vurture, G. W. , Sedlazeck, F. J. , Nattestad, M. , Underwood, C. J. , Fang, H. , Gurtowski, J. , & Schatz, M. C. (2017). GenomeScope: Fast reference‐free genome profiling from short reads. Bioinformatics, 33, 2202–2204. 28369201 10.1093/bioinformatics/btx153 PMC5870704

[ece34778-bib-0050] Xu, D. , Lou, B. , Zhou, W. , Chen, R. , Zhan, W. , & Liu, F. (2017). Genetic diversity and population differentiation in the yellow drum *Nibea albiflora* along the coast of the China Sea. Marine Biology Research, 13, 456–462.

[ece34778-bib-0051] Zhou, Y. , Holmseth, S. , Guo, C. , Hassel, B. , Höfner, G. , Huitfeldt, H. S. , … Danbolt, N. C. (2012). Deletion of the γ‐aminobutyric acid transporter 2 (GAT2 and SLC6A13) gene in mice leads to changes in liver and brain taurine contents. The Journal of Biological Chemistry, 287, 35733–35746. 22896705 10.1074/jbc.M112.368175 PMC3471754

