# Supplementary material for: Near‐complete genome assembly and annotation of the yellow drum (Nibea albiflora) provide insights into population and evolutionary characteristics of this species
Source: Ecol Evol. 2018 Dec 11;9(1):568–75. doi: 10.1002/ece3.4778 (PMC6342179; doi:10.1002/ece3.4778)
Supplement: Supplementary file 1 [file ECE3-9-568-s001.doc]

**Supplementary Figures and Tables**

**Figure S1.** 29-mer distribution of using jellyfish with 300 bp paired-end whole genome sequencing data.

***Table S1****. The information of repeat motif, primer sequences of 11 SSRs used in homozygous validation in N. albiflora.*

| Locus | Repeat motif | Forward primers | Reverse primers |
| --- | --- | --- | --- |
| YD-01 | (TG)10 | CTTGGTTTTAGGTATCCAGGCTCT | TGCCCACTCACCTGTGTCATT |
| YD-02 | (CA)10 | GCACACCTGACCGTGAACA | GAAGAGAACAACCTGGTAATGAACT |
| YD-03 | (TC)26 | TGCCTTTTGGAAATCAGCCT | ACATCTCCCTAACAGTCGCATAATA |
| YD-04 | （CA)6TA(CA)6 | CAACGCATGTCAGATTGGAG | CGCAACTCAGCACAGGATTA |
| YD-05 | (TG)13 | ACTCCAAGGCAGAGCTGACT | CATACCAGGATGCCGATCAT |
| YD-06 | (AC)9 | AGAGGCAGCCTCCATTCC | CCCACATGGTGTGTGCTTAG |
| YD-07 | (AC)11 | AGAGGAAAAGTGTGGCACATAAG | CGGGTTTACCCTCCACTGTA |
| YD-08 | (GA)15 | AAGACGGTATGCCAGGGTT | GTTCAGGAGCAAAAAAATAAGAGA |
| YD-09 | (CACT)7 | CCACATCCCAGTGCCAGTAAA | CTCGGCGCTCATAGGTAAAG |
| YD-10 | (CA)17 | CGACACCTCACACTTTCTTTTCTTA | TCCTTTATCCAGAAGCAGAAACTCG |
| YD-11 | (CT)25 | ACAGAACACATCCAAACAAAGG | AACCAAAAGAAAGGAGCGAC |

**Table S2.** Genome estimation using GenomeScope with k-mer of 17, 21, 25 and 29.

| K-mer | Genome size (Mb) | Repeat length (Mb) | Heterozygosity (%) |
| --- | --- | --- | --- |
| 17 | 573.2 | 167.1 | 0.252 |
| 21 | 577.5 | 55.9 | 0.274 |
| 25 | 579.6 | 48.5 | 0.272 |
| 29 | 581.0 | 45.6 | 0.268 |

**Table S3.** Summary statistics of Benchmarking Universal Single-Copy Orthologs (BUSCO) analysis for *N. albiflora* genome.

| BUSCO categories | Gene number | Percentage (%) |
| --- | --- | --- |
| Complete | 4,478 | 97.7 |
| Complete single-copy | 4,362 | 95.2 |
| Complete duplicate | 116 | 2.5 |
| Fragmented | 39 | 0.9 |
| Missing BUSCOs | 67 | 1.4 |

**Table S4.** Expanded and contracted gene families of *N. albiflora* genome.

| Type | Clustering | Family | Description | chicken | human | croaker | drum | stickle back | tilapia | medaka | platyfish | fugu | tetraodon | zebrafish | spotted fish |
| --- | --- | --- | --- | --- | --- | --- | --- | --- | --- | --- | --- | --- | --- | --- | --- |
| Expansion gene | OCG7 | Myosin | Myosin | 16 | 15 | 12 | 33 | 22 | 23 | 25 | 18 | 17 | 22 | 25 | 16 |
|  | OCG8 | Pkinase_Tyr | Protein tyrosine kinase | 14 | 13 | 10 | 21 | 21 | 21 | 20 | 23 | 21 | 24 | 20 | 14 |
|  | OCG10 | ANF_receptor | Receptor family ligand binding region | 1 | 1 | 0 | 14 | 11 | 31 | 19 | 9 | 21 | 12 | 54 | 29 |
|  | OCG13 | SNF | Sodium: neurotransmitter symporter family | 7 | 6 | 11 | 22 | 13 | 22 | 15 | 17 | 23 | 19 | 16 | 12 |
|  | OCG16 | ANF_receptor | Receptor family ligand binding region | 8 | 6 | 7 | 17 | 15 | 16 | 14 | 15 | 17 | 14 | 12 | 9 |
|  | OCG24 | 7tm_3 | 7 transmembrane sweet-taste receptor of 3 GCPR | 7 | 7 | 6 | 15 | 15 | 15 | 15 | 14 | 13 | 13 | 11 | 8 |
|  | OCG37 | Pkinase | Protein kinase domain | 6 | 3 | 4 | 12 | 11 | 10 | 11 | 11 | 11 | 12 | 12 | 6 |
|  | OCG43 | Anoctamin | Calcium-activated chloride channel | 6 | 5 | 5 | 11 | 11 | 11 | 7 | 12 | 8 | 10 | 9 | 8 |
|  | OCG50 | Tubulin | Tubulin/FtsZ family, GTPase domain | 6 | 15 | 1 | 9 | 8 | 9 | 8 | 7 | 7 | 6 | 9 | 7 |
|  | OCG54 | zf-C3HC4_2 | Zinc finger, C3HC4 type (RING finger) | 1 | 1 | 2 | 9 | 7 | 31 | 2 | 1 | 1 | 1 | 27 | 7 |
|  | OCG57 | Ion_trans | Ion transport protein | 5 | 6 | 1 | 10 | 7 | 10 | 8 | 10 | 9 | 8 | 8 | 5 |
|  | OCG59 | G-alpha | G-protein alpha subunit | 7 | 6 | 2 | 9 | 9 | 9 | 7 | 9 | 8 | 5 | 8 | 5 |
|  | OCG96 | Na_Ca_ex | Sodium/calcium exchanger protein | 4 | 3 | 1 | 8 | 6 | 7 | 6 | 7 | 7 | 7 | 7 | 4 |
|  | OCG97 | VWD | von Willebrand factor type D domain | 8 | 4 | 5 | 10 | 6 | 4 | 6 | 3 | 4 | 3 | 6 | 8 |
|  | OCG180 | Ion_trans | Ion transport protein | 4 | 3 | 3 | 9 | 2 | 6 | 3 | 7 | 5 | 5 | 5 | 3 |
|  | OCG998 | FIIND | Function to find | 1 | 7 | 0 | 3 | 0 | 1 | 0 | 1 | 0 | 0 | 9 | 4 |
|  | OCG1134 | Astacin | Astacin (Peptidase family M12A) | 2 | 1 | 1 | 4 | 3 | 2 | 4 | 0 | 4 | 0 | 2 | 1 |
|  | OCG1952 | 7tm_1 | 7 transmembrane receptor (rhodopsin family) | 0 | 0 | 0 | 9 | 0 | 1 | 0 | 3 | 0 | 0 | 2 | 3 |
|  | OCG1968 | 7tm_1 | 7 transmembrane receptor (rhodopsin family) | 1 | 0 | 1 | 4 | 2 | 3 | 1 | 0 | 3 | 1 | 1 | 1 |
|  | OCG1976 | Serpin | Serpin (serine protease inhibitor) | 0 | 1 | 0 | 3 | 2 | 1 | 1 | 1 | 3 | 1 | 4 | 1 |
|  | OCG2111 | V-set | Immunoglobulin V-set domain | 1 | 0 | 1 | 3 | 0 | 5 | 0 | 1 | 0 | 0 | 5 | 1 |
|  | OCG2151 | DDE_Tnp_1_7 | Transposase IS4 | 0 | 4 | 0 | 3 | 0 | 1 | 0 | 1 | 0 | 0 | 0 | 8 |
|  | OCG2250 | ApoL | Apolipoprotein L | 0 | 0 | 1 | 4 | 2 | 3 | 1 | 1 | 1 | 1 | 1 | 1 |
|  | OCG2271 | C1-set | Immunoglobulin C1-set domain | 0 | 0 | 1 | 5 | 1 | 0 | 6 | 0 | 0 | 1 | 1 | 1 |
|  | OCG2278 | C2 | C2 domain | 0 | 0 | 0 | 3 | 2 | 1 | 1 | 1 | 3 | 1 | 4 | 0 |
|  | OCG2444 | 7tm_1 | 7 transmembrane receptor (rhodopsin family) | 0 | 0 | 0 | 3 | 0 | 1 | 2 | 0 | 0 | 0 | 0 | 9 |
|  | OCG2558 | 7tm_1 | 7 transmembrane receptor (rhodopsin family) | 0 | 0 | 0 | 6 | 1 | 1 | 0 | 1 | 0 | 0 | 4 | 1 |
|  | OCG2896 | Chitin_synth_2 | Chitin synthase | 0 | 0 | 1 | 3 | 3 | 0 | 0 | 1 | 0 | 0 | 4 | 1 |
|  | OCG2905 | DDE_4 | DDE superfamily endonuclease | 0 | 0 | 1 | 6 | 2 | 3 | 0 | 0 | 0 | 0 | 0 | 1 |
|  | OCG3762 | V-set | Immunoglobulin V-set domain | 0 | 0 | 1 | 7 | 1 | 2 | 0 | 0 | 0 | 1 | 0 | 0 |
|  | OCG3770 | Ig_2 | Immunoglobulin domain | 0 | 0 | 2 | 4 | 1 | 4 | 1 | 0 | 0 | 0 | 0 | 0 |
|  | OCG9646 | 7tm_1 | 7 transmembrane receptor (rhodopsin family) | 0 | 0 | 0 | 3 | 0 | 3 | 0 | 1 | 0 | 0 | 1 | 2 |
|  | OCG11229 | SRCR | Scavenger receptor cysteine-rich domain | 0 | 0 | 1 | 3 | 1 | 4 | 0 | 0 | 0 | 0 | 0 | 0 |
|  | OCG11235 | Pkinase | Protein kinase domain | 0 | 0 | 1 | 6 | 0 | 0 | 0 | 0 | 0 | 0 | 2 | 0 |
|  | OCG11236 | missing | - | 0 | 0 | 0 | 3 | 1 | 0 | 2 | 1 | 1 | 0 | 0 | 1 |
|  | OCG11238 | missing | - | 0 | 0 | 2 | 5 | 0 | 0 | 1 | 1 | 0 | 0 | 0 | 0 |
|  | OCG12489 | LRR_6 | Leucine Rich repeat | 0 | 0 | 0 | 6 | 0 | 0 | 1 | 0 | 0 | 1 | 0 | 0 |
|  | OCG12491 | missing | - | 2 | 0 | 0 | 4 | 0 | 0 | 0 | 0 | 0 | 0 | 1 | 1 |
|  | OCG12493 | missing | - | 0 | 0 | 1 | 4 | 1 | 0 | 1 | 1 | 0 | 0 | 0 | 0 |
|  | OCG12494 | missing | - | 0 | 0 | 1 | 4 | 0 | 0 | 0 | 1 | 0 | 0 | 1 | 1 |
|  | OCG12495 | Ig_2 | Immunoglobulin domain | 0 | 0 | 1 | 4 | 0 | 2 | 0 | 0 | 0 | 1 | 0 | 0 |
|  | OCG13643 | V-set | Immunoglobulin V-set domain | 0 | 0 | 1 | 4 | 0 | 2 | 0 | 0 | 0 | 0 | 0 | 0 |
|  | OCG14321 | missing | - | 0 | 0 | 1 | 3 | 0 | 0 | 1 | 0 | 0 | 1 | 0 | 1 |
|  | OCG14615 | 7tm_1 | 7 transmembrane receptor (rhodopsin family) | 0 | 0 | 0 | 3 | 0 | 0 | 2 | 0 | 0 | 1 | 0 | 0 |
| Contraction gene | OCG2 | NACHT | NACHT domain | 0 | 0 | 4 | 1 | 2 | 159 | 8 | 17 | 15 | 11 | 334 | 14 |
|  | OCG17 | I-set | Immunoglobulin I-set domain | 6 | 5 | 45 | 10 | 10 | 11 | 9 | 11 | 11 | 18 | 7 | 7 |
|  | OCG38 | Ldl_recept | Low-density lipoprotein receptor | 6 | 6 | 23 | 8 | 7 | 8 | 6 | 11 | 9 | 8 | 9 | 7 |
|  | OCG47 | SPRY | SPRY domain | 0 | 2 | 2 | 0 | 36 | 21 | 2 | 2 | 2 | 2 | 19 | 10 |
|  | OCG150 | Lipoxygenase | Lipoxygenase | 1 | 7 | 4 | 2 | 4 | 7 | 8 | 6 | 8 | 3 | 6 | 3 |
|  | OCG217 | SPEC | Spectrin repeat | 3 | 2 | 13 | 3 | 4 | 4 | 4 | 3 | 5 | 5 | 4 | 2 |
|  | OCG452 | Crystall | Beta/Gamma crystallin | 0 | 4 | 3 | 0 | 4 | 4 | 4 | 1 | 2 | 4 | 4 | 10 |
|  | OCG1086 | C1q | C1q domain | 0 | 0 | 2 | 0 | 2 | 14 | 0 | 2 | 1 | 0 | 4 | 0 |
|  | OCG1369 | RVT_1 | Reverse transcriptase (RNA-dependent DNA polymerase) | 0 | 0 | 2 | 0 | 0 | 2 | 2 | 2 | 0 | 0 | 14 | 0 |
|  | OCG1714 | DDE_1 | DDE superfamily endonuclease | 0 | 0 | 2 | 0 | 3 | 9 | 1 | 2 | 0 | 0 | 3 | 0 |
|  | OCG3109 | C1q | C1q domain | 0 | 0 | 2 | 0 | 0 | 2 | 1 | 4 | 0 | 1 | 3 | 0 |
